# Adduct of the blistering warfare agent sesquimustard with human serum albumin and its mass spectrometric identification for biomedical verification of exposure

**DOI:** 10.1007/s00216-020-02917-w

**Published:** 2020-09-09

**Authors:** Marc-Michael Blum, Annika Richter, Markus Siegert, Horst Thiermann, Harald John

**Affiliations:** 1 Blum – Scientific Services, Björnsonweg 70d, 22587 Hamburg, Germany; 2 Department of Chemistry, Humboldt-Universität zu Berlin, Brook-Taylor-Straße 2, 12489 Berlin, Germany; 3 Bundeswehr Institute of Pharmacology and Toxicology, Neuherbergstraße 11, 80937 Munich, Germany

**Keywords:** Hydroxyethylthioethyl, Protein adduct, Sulfur mustard, Verification, Vesicant

## Abstract

Apart from the well-known sulfur mustard (SM), additional sulfur-containing blistering chemical warfare agents exist. Sesquimustard (Q) is one of them and five times more blistering than SM. It is a common impurity in mustard mixtures and regularly found in old munitions but can also be used in pure form. Compared to the extensive literature on SM, very little experimental data is available on Q and no protein biomarkers of exposure have been reported. We herein report for the first time the adduct of Q with the nucleophilic Cys^34^ residue of human serum albumin (HSA) formed in vitro and introduce two novel bioanalytical procedures for detection. After proteolysis of this HSA adduct catalyzed either by pronase or by proteinase K, two biomarkers were identified by high-resolution tandem mass spectrometry (MS/HR MS), namely a dipeptide and a tripeptide, both alkylated at their Cys residue, which we refer to as HETETE-CP and HETETE-CPF. HETETE represents the Q-derived thio-alkyl moiety bearing a terminal hydroxyl group: “hydroxyethylthioethylthioethyl.” Targeting both peptide markers from plasma, a micro liquid chromatography–electrospray ionization tandem mass spectrometry method working in the selected reaction monitoring mode (μLC-ESI MS/MS SRM) was developed and validated as well suited for the verification of exposure to Q. Fulfilling the quality criteria defined by the Organisation for the Prohibition of Chemical Weapons, the novel methods enable the detection of exposure to Q alone or in mixtures with SM. We further report on the relative reactivity of Q compared to SM. Based on experiments making use of partially deuterated Q as the alkylating agent, we rule out a major role for six-membered ring sulfonium ions as relevant reactive species in the alkylation of Cys^34^. Furthermore, the results of molecular dynamics simulations are indicative that the protein environment around Cys^34^ allows adduct formation with elongated but not bulky molecules such as Q, and identify important hydrogen bonding interactions and hydrophobic contacts.

**Electronic supplementary material:**

The online version of this article (10.1007/s00216-020-02917-w) contains supplementary material, which is available to authorized users.

**Figure:**
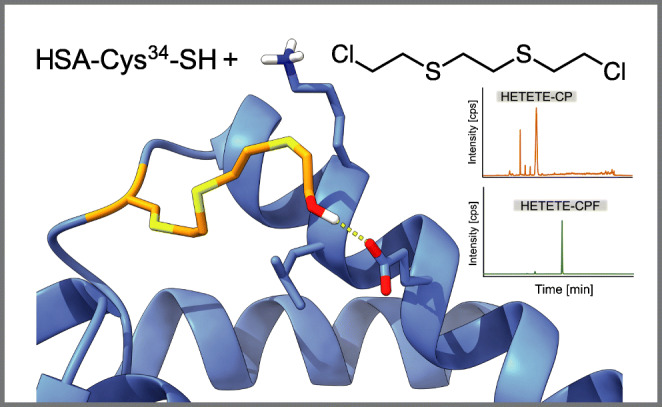
Graphical abstract

## Introduction

**Fig. 1:**
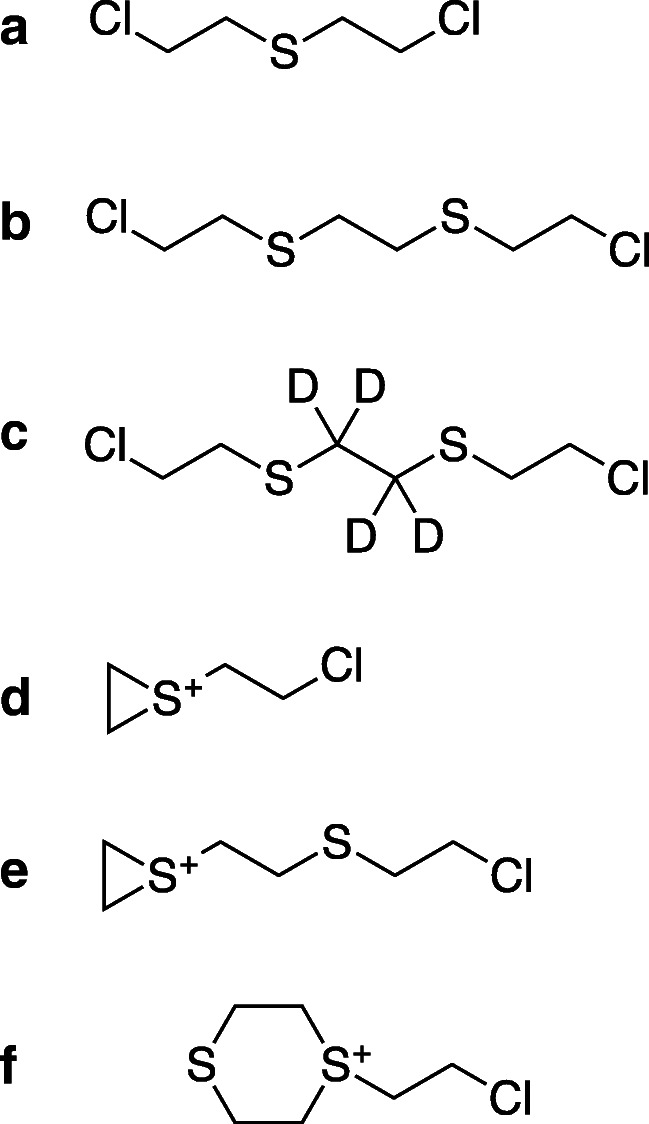
Chemical structures of blister agents and their reactive intermediates: **a** sulfur mustard (SM), **b** sesquimustard (Q), **c** sesquimustard-*d*_4_, **d** reactive episulfonium ions of SM, **e** reactive episulfonium ions of Q, and **f** the six-membered ring sulfonium ion of Q

Vesicants, chemicals that cause blistering of the skin, have been used as warfare agents for more than 100 years. The most prominent member of this group is sulfur mustard (SM, bis(2-chloroethyl) sulfide, CAS 505-60-2, Fig. 1a) [1, 2]. It has been employed as recently as 2015 and 2016 by the terrorist group “Islamic State” in the Syrian Arab Republic and northern Iraq [3]. SM causes painful blisters and erythema on exposed skin areas characterized by delayed and complicated wound healing and shows a complex toxicokinetic behavior [1, 4].

Verification of exposure is an important capability in order to guide medical therapy of casualties but also for generating evidence that a violation of the international norm on the nonuse of chemical weapons has occurred [5]. This norm is laid down in the Chemical Weapons Convention (CWC), which entered into force in 1997, and is implemented by the Organisation for the Prohibition of Chemical Weapons (OPCW) [6]. The OPCW designates laboratories for the analysis of authentic biomedical samples through successful participation in biomedical proficiency tests (BioPTs). In these BioPTs, samples of biological fluids spiked with SM represent a regular analytical challenge. The most long-lasting in vivo biomarkers of exposure are protein-adducts such as those formed with human serum albumin (HSA). HSA-adducts result from covalent linkage of SM to, e.g., the Cys^34^ residue, and have emerged as the most prominent biomarkers [7–10]. They have been used on several occasions for the analysis of samples from OPCW interlaboratory exercises but also from exposed victims [11–14].

However, SM is not the only blistering agent causing a serious threat for military personnel as well as for civilians. Among the chemicals listed in the Annex on Chemicals of the CWC are a number of higher sulfur mustards, including sesquimustard (Q, Fig. 1b).

Q (1,2-bis(2-chloroethylthio) ethane, CAS 3563-36-8, Fig. 1b), a white solid (melting point 56.6 °C), is about five times more blistering than SM [15]. As a solid, it represents a very persistent contact hazard but has so far not been used in its pure form. It was produced as a component in the British HQ process during World War II resulting in a 70:30 w/w mixture of SM:Q [16]. It is also regularly found in old distilled (HD) and Levinstein (H) mustard. For example, a ton container of Levinstein mustard sampled in the USA after 60 years of storage before destruction contained about 10% w/w of Q [17].

Despite its relevance, the number of scientific publications on Q is small, especially when compared with the extensive literature on SM. Q was first reported by Bennett and Whincop in 1921 [18]. Its toxic properties but also potential use as an alternative to nitrogen mustard anti-tumor drugs were studied and reported in a few publications thereafter [15, 19, 20]. St. Quintin et al. reported on the hydrolysis of Q observing six-membered ring sulfonium ions (Fig. 1f) as long-lived reaction intermediates in addition to the three-membered ring episulfonium ions known from SM (Fig. 1e) adding important insight into the behavior of Q in aqueous systems [21]. Analysis of Q and its hydrolysis and oxidation products from environmental samples by means of gas and liquid chromatography (LC) coupled to mass spectrometry (MS) has been described [22–26]. A method for the quantification of the urinary biotransformation products of Q using LC coupled to tandem mass spectrometry (MS/MS) was also published [27]. However, no methods describing protein biomarkers and their analysis have been presented so far.

We herein report on the adduct of Q formed with HSA upon reaction of the agent with the Cys^34^ residue of the protein. We further investigated the effect of adduct formation on the protein structure of HSA with a special focus on the local protein environment around Cys^34^ using molecular dynamics (MD) simulations as well as the potential role of six-membered ring sulfonium ions as reactive species. In analogy to the S-linked hydroxyethylthioethyl-adduct (HETE-HSA) found with SM as the reaction partner [8, 10], we refer to the adduct formed with Q as HETETE-HSA (“hydroxyethylthioethylthioethyl”).

## Materials and methods

### Chemicals and reagents

Acetone (p.A.), acetonitrile (ACN, gradient grade), CH_2_Cl_2_ (for GC), isopropanol (iPrOH, p.A), water (LiChrosolv), and proteinase K (recombinant from *Pichia pastoris*, PCR grade) were purchased from Merck (Darmstadt, Germany); formic acid (FA ≥ 98%) and NaOCl solution for decontamination (12% Cl_2_) were from Carl Roth (Karlsruhe, Germany); pronase from *Streptomyces griseus* was from Roche (lot no. 70327222, Mannheim, Germany); NH_4_HCO_3_ (ultra-grade, ≥ 99.5%) was from Fluka (Buchs, Switzerland); and three-fold deuterated atropine (Atr-*d*_3_) was from CDN Isotopes (Pointe Claire, Quebec, Canada). Pooled human ethylenediaminetetraacetic acid (EDTA) plasma was purchased from Dunn Labortechnik (Asbach, Germany) and human EDTA plasma from different individuals was purchased from Sonnen-Gesundheitszentrum (Munich, Germany). SM was made available by the German Ministry of Defence and tested for integrity and purity (99%) in-house by NMR spectroscopy.

The Q hydrolysis product 1,2-bis(2-hydroxyethylthio) ethane and its partially deuterated form 1,2-bis(2-hydroxyethylthio)-*d*_4_-ethane have been synthesized by one of the authors during his tenure at the OPCW laboratory following methods from the scientific literature [28]. Both diols were chlorinated to yield Q and its partially deuterated variant in pure form (Fig. 1b and c). Molecular structure and purity (> 95%) were confirmed by ^1^H- and ^13^C-NMR spectroscopy.

1,2-Bis(2-chloroethylthio)ethane: ^1^H-NMR (CDCl_3_, 400 MHz): *δ* 2.81 (s, 4H), 2.92 (t, 4H, *J* = 6.61 Hz), 3.64 (t, 4H, *J* = 6.61 Hz). C {H}-NMR (CDCl_3_, 100 MHz): *δ* 32.70, 34.53, 43.07.

1,2-Bis(2-chloroethylthio)-*d*_4_-ethane: ^1^H-NMR (CDCl_3_, 400 MHz): *δ* 2.91 (t, 4H, *J* = 6.52 Hz), 3.66 (t, 4H, *J* = 6.52 Hz). C {H}-NMR (CDCl_3_, 100 MHz): *δ* 32.68*, 34.53, 43.07.

*Signal intensity significantly reduced compared to the nondeuterated compound.

Stock solutions of SM and Q (150 mM each) were prepared in CH_2_Cl_2_ and further diluted with iPrOH to yield working solutions (5 mM).

#### Caution

*SM and Q are potent vesicants and should only be handled by experienced and trained personnel using appropriate protective equipment in a properly working fume hood also requiring strict decontamination of all materials the poisons had contact to.*

### Incubation of plasma with Q and SM

References of HSA-adducts formed with Q, Q-*d*_4_, and SM were produced by mixing human EDTA plasma with the working solutions of the respective agent yielding 100 μM each. Incubations were carried out for 2 h at 37 °C and subsequently overnight at 4 °C. Samples were stored at − 25 °C until further processing.

### Plasma sample preparation for HETETE-CP and HETETE-CPF analysis

Plasma samples (50 μL) and blank plasma (50 μL) were mixed with 250 μL acetone for protein precipitation followed by rigorous vortexing and centrifugation (3500 *g*, 3 min at room temperature, RT). The protein pellet was washed with 250 μL acetone supported by ultrasonication for 2 min and final centrifugation (3500 *g*, 3 min, RT). The supernatant was discarded and the pellet air dried. Subsequently, 200 μL 50 mM NH_4_HCO_3_ and 50 μL pronase (10 mg/mL in 50 mM NH_4_HCO_3_ to generate HETETE-CP) or 50 μL proteinase K solution (20 mg/mL in 50 mM NH_4_HCO_3_ to generate HETETE-CPF) were added for dissolution and proteolysis (2 h at 50 °C). Afterwards, 750 μL ACN was added for protein precipitation and centrifugation (13,000 *g*, 5 min, RT). The supernatant was dried under reduced pressure and redissolved in 250 μL 50 mM NH_4_HCO_3_. A portion of 30 μL was mixed with 60 μL Atr-*d*_3_ solution (3 ng/mL in 0.5% v/v FA) for micro liquid chromatography–electrospray ionization tandem-mass spectrometry working in the selected reaction monitoring mode (μLC-ESI MS/MS SRM).

### μLC-ESI MS/MS (SRM) analysis

Chromatography was carried out using a M5 microLC system comprising an integrated autosampler (15 °C) allowing 20 μL sample volume injection (AB Sciex, Darmstadt, Germany). Peptides were separated at 60 °C on an Acquity UPLC HSS T3 column (50 × 1.0 mm I.D., 1.8 μm, 100 Å; Waters, Eschborn, Germany) protected by a precolumn (Security Guard Ultra cartridge C18 peptide; Phenomenex, Aschaffenburg, Germany) applying a binary gradient mobile phase with 30 μL/min of solvent A (0.05% v/v FA) and solvent B (ACN/H_2_O 80:20 v/v, 0.05% v/v FA): *t* [min]/B [%]: 0/2, 11/60, 11.5/95, 13.5/95, 14/2, 15/2. Using an ESI interface working in positive mode (5 kV) the QTrap 6500^+^ mass spectrometer (AB Sciex) was coupled to monitor product ions after collision-induced dissociation (CID) of analytes using nitrogen as collision gas. The following MS settings were applied: declustering potential (DP) 60 V, curtain gas (CUR) 30 psi (2.07 × 10^5^ Pa), temperature 200 °C, source gas 1 (GS1) 50 psi (3.45 × 10^5^ Pa), source gas 2 (GS2) 60 psi (4.14 × 10^5^ Pa), entrance potential (EP) 10 V, cell exit potential (CXP) 10 V each, and dwell time 50 ms. The single protonated precursor ions of the biomarkers were fragmented with a collision energy (CE) of 30 V to generate product ions by MS/MS working in the SRM mode: HETETE-CP: *m*/*z* 383.1 > *m*/*z* 105.0, *m*/*z* 217.1; HETETE-CPF: *m*/*z* 530.2 > *m*/*z* 105.0, *m*/*z* 137.0; HETE-CP: *m*/*z* 323.1 > *m*/*z* 105.0, *m*/*z* 137.0; and HETE-CPF: *m*/*z* 470.2 > *m*/*z* 105.0, *m*/*z* 137.0. The internal standard Atr-*d*_3_ was monitored as follows: *m*/*z* 293.3 > *m*/*z* 127.1 and *m*/*z* 93.1 using a CE of 42 V.

### μLC-ESI MS/HR MS (Orbitrap) analysis

Initial high-resolution tandem mass spectrometric (MS/HR MS) detection of HETETE-adducts was carried out to determine the exact masses of product ions. For chromatography of a 20-μL sample volume, a MicroPro pump (Eldex Laboratories, Napa, CA, USA) in combination with an INTEGRITY autosampler and a MISTRAL column oven (both Spark Holland, Emmen, The Netherlands) was used. The stationary and mobile phases were the same as described above, but the gradient was as follows: *t* [min]/B [%]: 0/10; 11/60; 11.1/95; 13.9/95; 14/10; 15/10. The system was controlled by the Eldex MicroPro 1.0.54 software (Eldex Laboratories) and coupled on-line to a QExactive plus Orbitrap mass spectrometer via the HESI II ion source (Thermo Scientific, Bremen, Germany). The following MS parameters were applied to monitor product ions of the single protonated precursor ions of HETETE-CP (*m*/*z* 383.1), *d*_4_-HETETE-CP (*m*/*z* 387.1), HETETE-CPF (*m*/*z* 530.2), and *d*_4_-HETETE-CPF (*m*/*z* 534.2) in the parallel reaction monitoring (PRM) mode with a mass spectrometric resolution of 35,000 FWHM (full width at half maximum at *m*/*z* 200): sheath gas flow 23 arbitrary units (a.u.), aux gas flow 8 a.u., sweep gas flow 1 a.u., spray voltage 3.5 kV, capillary temperature 250 °C, S-lens RF level 50 a.u., and aux gas heater temperature 125 °C. Data were acquired between 3 and 15 min. The maximum automated gain control value (AGC) was set to 2 × 10^5^ charges in the Orbitrap analyzer, the maximum injection time (IT) to 100 ms, the isolation window to 2.0 *m*/*z*, the isolation offset to 0.5 *m*/*z*, and a fixed first mass to 50.0 *m*/*z*. For all analytes, a normalized collision energy (NCE, normalized to *m*/*z* 200, *z* = 1) of 25 V was applied. The MS system was controlled by the Excalibur 4.1 software (Thermo Scientific), and for data processing, the FreeStyle 1.3 software was used (Thermo Scientific).

### Selectivity

The selectivity of HETETE-CP and HETETE-CPF detection in plasma samples was investigated by μLC-ESI MS/MS (SRM) analysis of blank plasma (not exposed to any agent) from 6 individuals following the standard protocol for interference detection.

### Determination of linear range and lower limit of identification

To determine the linearity of HETETE-CP and HETETE-CPF peak areas plotted against the concentration of the agent applied for incubation with plasma and to estimate the respective lower limits of identification (LOI), 12 plasma standards were produced. Accordingly, the working solution of Q was diluted in iPrOH yielding 12 differently concentrated solutions. An aliquot of 20 μL was added to 1980 μL plasma each (*n* = 3) resulting in Q concentrations of 50 μM, 20 μM, 4 μM, 800 nM, 400 nM, 160 nM, 80 nM, 32 nM, 16 nM, 6.4 nM, 3.2 nM, and 1.28 nM. Samples were incubated in triplicate, prepared, and analyzed by μLC-ESI MS/MS (SRM) as described above. The LOI was defined as the lowest concentration of the spiked agent still allowing detection of the adduct in all three replicates exhibiting the respective ion ratios of product ions obtained from a reference sample (50 μM Q in plasma).

### Stability of HETETE-CP and HETETE-CPF in the autosampler

The stability of both peptide-adducts present in the prepared plasma samples stored at 15 °C in the autosampler was determined hourly by μLC-ESI MS/MS (SRM) analysis over a 24-h period. Peak areas obtained from the extracted ion chromatograms (XIC) of the most intense product ions (*m*/*z* 105.0 each) were used to follow the relative concentration–time profiles.

### Freeze and thaw cycles

Plasma was incubated with Q as described above in concentrations of 50 μM and 20 μM. Three aliquots (500 μL each) of both mixtures were pipetted into separate reaction vials, and 50 μL each was analyzed immediately (day 0, *n* = 3) by μLC-ESI MS/MS (SRM) to monitor HETETE-CP following the standard protocol. The remaining volumes were frozen and stored at − 20 °C for 24 h prior to thawing and repeated analysis (day 1, *n* = 3). This cycle of freezing and thawing was repeated three times (day 2 and day 3, *n* = 3, each). Peak areas obtained from XICs (*m*/*z* 105.0) were determined to follow the relative concentrations of HETETE-CP as a measure of HETETE-HSA stability.

### Co-incubation to characterize the relative reactivity of Q and SM

Buffered HSA solutions (960 μL, 133 μg/mL in PBS, 1.96 μM) were incubated in ultrafiltration (UF) devices (0.5 mL Amicon ultrafiltration unit, molecular weight cutoff 10 kDa, Merck-Millipore, Darmstadt, Germany) with (i) 100 μM SM, (ii) 100 μM Q, (iii) 100 μM SM plus 100 μM Q, and (iv) 100 μM SM plus 10 μM Q for 2 h at 37 °C. Aliquots of 100 μL were mixed with 300 μL 50 mM NH_4_HCO_3_ and 100 μL pronase solution (10 mg/mL in 50 mM NH_4_HCO_3_) carried out in triplicate. Following proteolysis for 2 h at 37 °C, samples were subjected to UF (10 min at 12,070 *g*, 15 °C) and portions of the filtrates (30 μL) were diluted 1:3 with Atr-*d*_3_ solution (3 ng/mL in 0.5% v/v FA) prior to μLC-ESI MS/MS (SRM) analysis. Peak areas of the XICs of the product ions at *m*/*z* 105.0 were determined as a measure of HETE-CP and HETETE-CP for relative quantification of the respective HSA-adducts.

### MD simulations

MD simulations were carried out using the GROMACS package [29] (v. 2020.2) employing the GROMOS96 53A7 force field [30]. The topology for cysteine adducted by Q (HETETE-Cys^34^) was created using the Automated Topology Builder (ATB) version 3.0 [31], and the resulting values (ATB Molecule ID 478917) were used to add the modified amino acid to the force field. Coordinates for HSA were obtained from the protein database (PDB entry: 1AO6) and used for the simulation of the apo protein. The starting structure for the construction of the HETETE-Cys^34^ variant of HSA was extracted from the production simulation (200 ns) of apo HSA and a frame was chosen in which Cys^34^ is solvent accessible and the HETETE moiety could be constructed without steric hindrance of neighboring residues. The Cys^34^ residue of HSA was modified using UCSF Chimera [32] so that the HETETE moiety points either into the solvent or fits into the groove between the two helices adjacent to Cys^34^. Apo HSA and variants were centered in a cubic unit cell with a distance between solute and box edge of 1.0 nm that was subsequently filled with simple point charge (SPC) water [33]. Fourteen sodium ions were added to balance the charge. Steepest descent minimization was performed, followed by 200 ps of canonical (NVT) equilibration and 200 ps of equilibration under an isothermal-isobaric (NPT) ensemble. Production simulations of 200 ns for apo HSA and 100 ns for the two HETETE-Cys^34^ variants followed. Positional restraints were applied to the protein during equilibration and released for the production run. All bond lengths were constrained using the LINCS method, allowing a 2-fs time step [34]. The Verlet cutoff scheme was applied [35]. Long-range electrostatic interactions were calculated with the particle mesh Ewald (PME) method [36, 37]. Simulations utilized the velocity-rescaling thermostat [38] and Parrinello–Rahman barostat [39, 40]. Further details can be found in the Electronic supplementary material (ESM).

## Results and discussion

The analysis of biomedical samples for possible exposure to SM is well established. While biotransformation products in urine are only present for a few days after exposure, protein-adducts can often be detected for several weeks [4]. Therefore, protein-adducts of SM are generally considered the most valuable biomarkers. As there are currently no established protein biomarkers of exposure to Q, a person exposed to this agent will show the typical clinical signs and symptoms of SM injury, but the biomedical analysis will turn out negative. Also, the co-exposure to SM and Q due to the presence of Q as a minor component of the mustard preparations will remain undetected. Therefore, protein biomarkers for exposure to Q must be identified and analytical procedures must be established following internationally accepted quality standards.

The OPCW tests laboratory capabilities through BioPTs. The regulations applicable for the reporting of data in these BioPTs also apply when reporting data from real samples collected on missions [41]. Positive identification of an agent is usually achieved through reporting of at least two different biomarkers where every biomarker is identified by an individual analytical method. Markers are divided into primary and secondary ones. While secondary markers are not unambiguous for identification but able to support identifications based on primary ones, data has to be reported from at least one primary biomarker. For the identification of SM in human plasma samples, the primary biomarkers are HETE-CP (from HSA), HETE-CPF (from HSA), N1/N3-HETE-His (from HSA and Globin), and other protein- or amino acid-adducts (e.g., AE^230^(-HETE) VSKL from HSA [12] and LGM^329^(-HETE) F from HSA, [9]), while the secondary marker for SM is thiodiglycol [41]. Our aim was to develop methods for the analysis and identification of HETETE-CP and HETETE-CPF as primary biomarkers of Q in plasma. Analysis of these two peptides will be sufficient to identify the spiking chemical Q in OPCW BioPTs and therefore also for the identification of exposure to Q in real samples. Accordingly, the formation of Q-adducts in plasma corresponding to those known from SM had to be detected and identified.

### Identification of HETETE-CP and HETETE-CPF by MS/HR MS

**Fig. 2:**
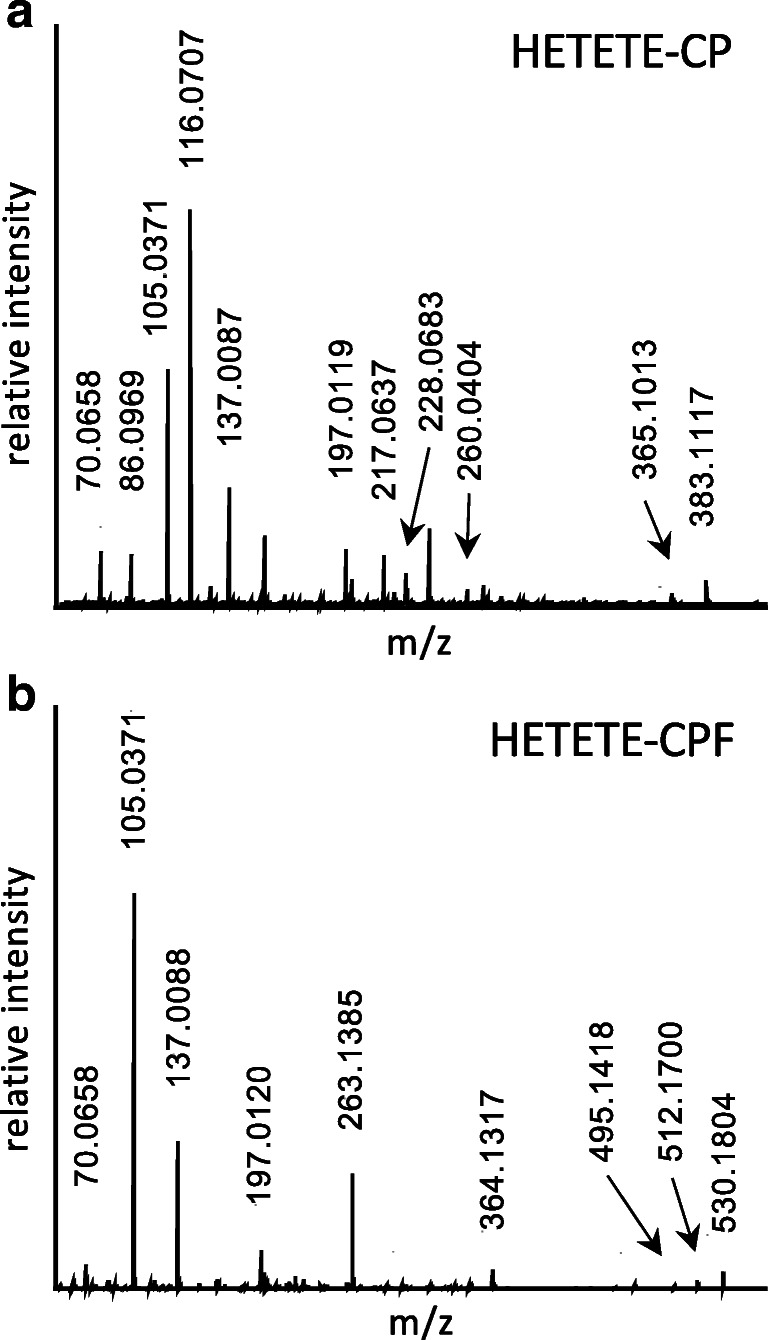
High-resolution product ion spectra of the alkylated di- and tripeptide biomarkers: **a** HETETE-CP, [M+H]^+^ *m*/*z* 383.117 and **b** HETETE-CPF, [M+H]^+^ *m*/*z* 530.1804. Spectra were extracted from μLC-ESI MS/HR MS (Orbitrap) analysis of HETETE-HSA-adducts after proteolysis with pronase (HETETE-CP) and proteinase K (HETETE-CPF). These references were produced by incubation of plasma with Q (50 μM). Labeled signals were assigned to product ions of the single protonated biomarkers as summarized in Table 1 and ESM Table S1

HETETE-CP and HETETE-CPF were identified based on MS/HR MS spectra extracted from chromatographic separation. Product ion spectra of both protonated biomarker precursor ions (HETETE-CP *m*/*z* 383.1 and HETETE-CPF *m*/*z* 530.2) are shown in Fig. 2 and the structural assignment of the signals is summarized in Table 1 for HETETE-CP. Assignments for HETETE-CPF can be found in the ESM (Table S1).

**Table 1:**
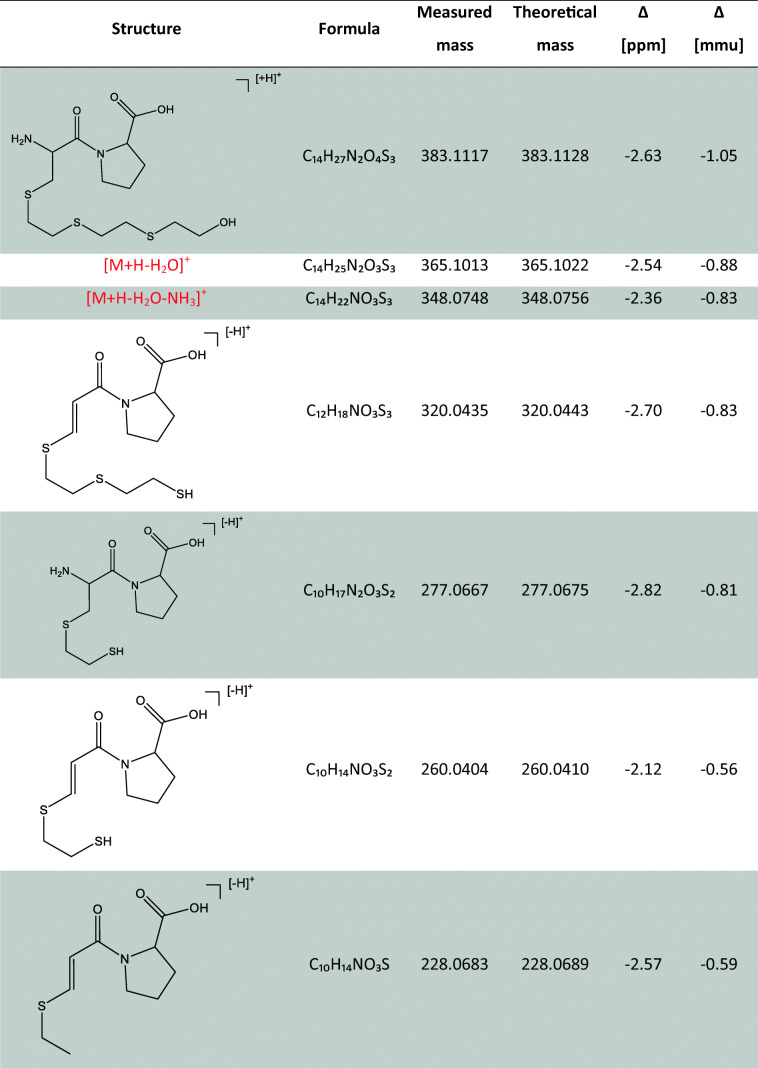

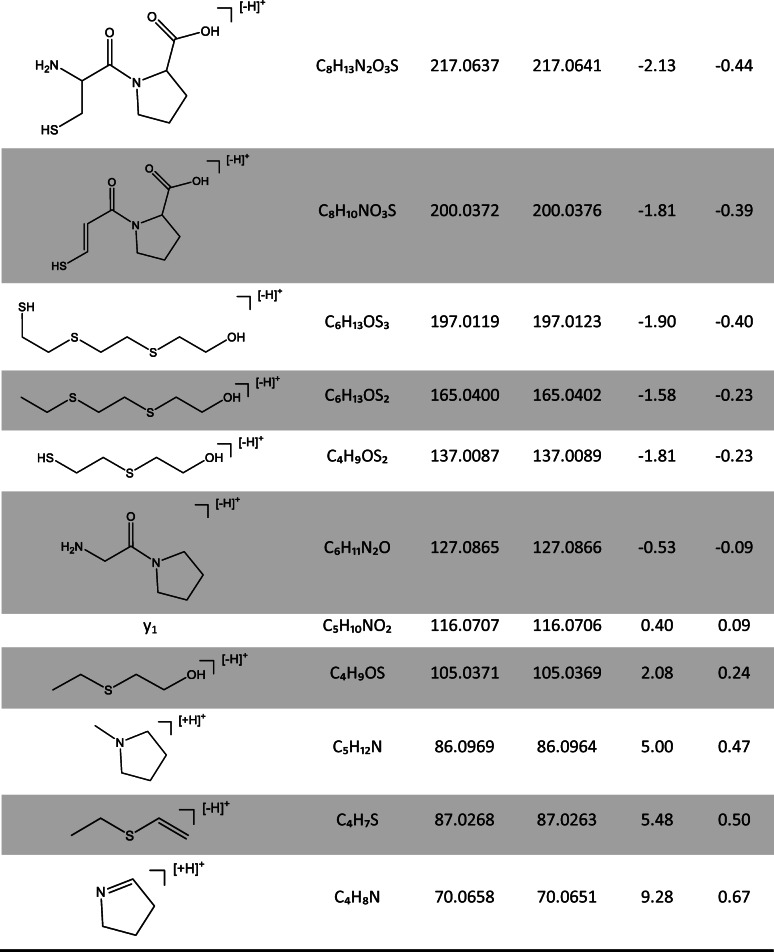
Product ions of single protonated HETETE-CP

Data was extracted from a μLC-ESI MS/HR MS (Orbitrap) run of HETETE-HSA-adducts after proteolysis with pronase. This reference was produced by incubation of plasma with Q (50 μM). The corresponding product ion spectrum is shown in Fig. 2a. Mass calculation was done using the FreeStyle 1.3 software (Thermo Fisher). Structures represent one possible isomer each

Characteristic predominant ions of HETETE-CP were found at *m*/*z* 105.0371 representing the HETE moiety and at *m*/*z* 137.0087 corresponding to the HETE moiety with the adjacent sulfur atom (Table 1). The specific product ion consisting of the dipeptide CP was found at *m*/*z* 217.0637 even though present in lower intensity (Fig. 2a). The CID of HETETE-CPF also yielded signals at about *m*/*z* 105.0 and *m*/*z* 137.0 indicating the presence of the HETETE moiety. In addition, product ions at *m*/*z* 263.1385 and *m*/*z* 364.1317 found with high intensity were derived from the peptide backbone of the biomarker and thus also confirmed the identity of HETETE-CPF. Interestingly, the agent-related product ions at *m*/*z* 105.0 and *m*/*z* 137.0 have also been reported before to be generated by CID of HETE-CP [7, 13] and HETE-CPF [10] representing the same chemical structures. Comparable product ions derived from similar cleavage sites of the thio-alkyl chain that represent the entire HETETE moiety either with (*m*/*z* 197.0119) or without (*m*/*z* 165.0400) the S*γ* of Cys^34^ were only detected with minor intensity (Fig. 2) not well suited for their use in μLC-ESI MS/MS (SRM) monitoring. The intensity of the product ion of HETETE-CP at *m*/*z* 116.0707 (Fig. 2a) was found to be influenced by interferences derived from the sample matrix despite exact mass determination and was therefore not appropriate for compound detection with optimal selectivity. Signals referred to above were assigned with a mass deviation typically not exceeding 3 ppm (Table 1, ESM Table S1), thus proving highest confidence for biomarker identification. Therefore, for optimum sensitivity and selectivity, the transitions to *m*/*z* 105.0 were chosen for both biomarkers in μLC-ESI MS/MS (SRM) analysis in addition to *m*/*z* 217.1 for HETETE-CP and to *m*/*z* 137.0 for HETETE-CPF. For the most sensitive detection of the product ions, a modern triple quadrupole mass spectrometer was used promising optimal sensitivity.

### Selectivity of μLC-ESI MS/MS (SRM) analysis

**Fig. 3:**
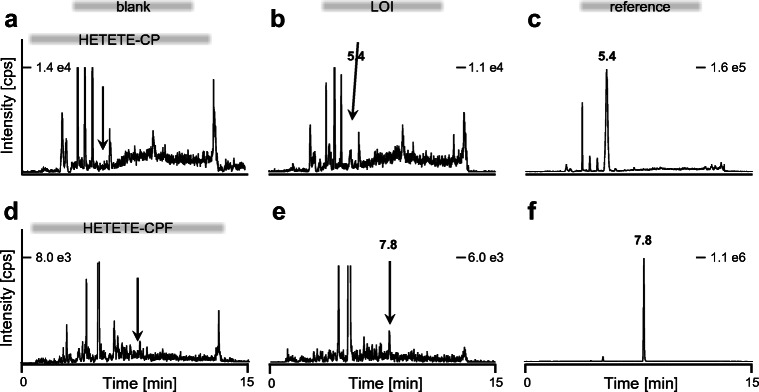
Detection of the alkylated di- and tripeptide biomarkers of Q exposure in human plasma. HETETE-CP: **a** blank, **b** plasma incubated with Q (32 nM) corresponding to the lower limit of identification (LOI), and **c** reference sample produced after plasma incubation with 100 μM Q. The alkylated dipeptide was produced by pronase cleavage of adducted HSA in plasma. HETETE-CPF: **d** blank, **e** plasma incubated with Q (6.4 nM) corresponding to the LOI, and **f** reference sample produced after plasma incubation with 50 μM Q. The alkylated tripeptide was produced by proteinase K cleavage of adducted HSA in plasma. All analyses were performed as μLC-ESI MS/MS runs in the selected reaction monitoring mode. For reasons of clarity, only the traces of the product ion at *m*/*z* 105.0 are illustrated for both biomarkers

Investigation of blank plasma from 6 individuals revealed that optimal detection of HETETE-CP was possible without any interferences when using the product ions at *m*/*z* 105.0 and *m*/*z* 217.1. Optimum monitoring of HETETE-CPF not showing any interferences was achieved using the transitions to *m*/z 105.0 and *m*/*z* 137.0 Therefore, product ions mentioned above were chosen to set up the verification method simultaneously detecting the alkylated di- and tripeptide. XICs of a blank plasma sample are shown in Fig. 3a and d illustrating the most intense product ion at *m*/*z* 105.0, each.

### Linear range and LOI

To characterize the Q concentration-dependent amount of the HETETE-Cys^34^-adduct of HSA, plasma was spiked with rising concentrations of Q to determine the HETETE-CP and HETETE-CPF peak areas obtained from the XICs of their product ions at *m*/*z* 105.0, each. The dipeptide-adduct (logP − 1.8593) was detected at *t*_R_ 5.4 min and the tripeptide-adduct (logP − 1.2455) at *t*_R_ 7.8 min corresponding to the higher hydrophobicity of HETETE-CPF. In both cases, linearity was found from 1.3 nM to 50 μM (*r*^2^ > 0.996) indicating dose-dependent adduct formation as well as reliable sample preparation procedures. The ion ratios of the diverse product ions (given in percentage) were calculated from the peak area (*A*) ratio of the relevant XICs, e.g., 100 × *A*(*m*/*z* 137.0)/*A*(*m*/*z* 105.0) abbreviated as (137/105). The following ion ratios were found for these transitions: HETETE-CP (217/105) 50.1% and HETETE-CPF (137/105) 53.3%. According to the OPCW quality criteria, ion ratios from biomarkers of any sample had to fit those of a reference for unambiguous identification. The LOI was defined as the lowest concentration of Q still fulfilling this criterion. Accordingly, the LOI for HETETE-CP detection was found at 32 nM Q in plasma, whereas 6.4 nM was found for HETETE-CPF. XICs (*m*/*z* 105.0) corresponding to these LOI concentrations are illustrated in Fig. 3b and e. These LOI are in the same range as those of the corresponding HSA-adducts of SM HETE-CP (156 nM and 19.5 nM [13]) and HETE-CPF (10 nM [10]). However, such limits strictly depend on the method-specific sample preparation procedures with respect to concentrating and diluting working steps as well as to instruments used in terms of injected sample amount, ion yield (sensitivity), and mass resolution (selectivity). Therefore, the mentioned LOI were quite similar for all vesicant biomarkers.

### Stability in the autosampler

HETETE-CP as well as HETETE-CPF were found to be of sufficient stability in the prepared samples when stored at 15 °C in the autosampler (results not shown). Only a slight peak area decrease of less than 7% for both biomarkers indicated a negligible concentration decrease within the 24-h test period thus documenting good stability beneficial especially when large sets of samples have to be analyzed.

### Freeze and thaw cycles

No degradation of HETETE-HSA in frozen and thawed plasma was determined by monitoring the HETETE-CP biomarker (data not shown), thus documenting that plasma samples can be handled following common rules of plasma sample treatment without the need for special care.

### Co-incubation to characterize the relative reactivity of SM and Q for Cys^34^ alkylation

The co-incubation experiment was carried out to investigate the relative reactivity of SM and Q toward alkylation of Cys^34^ in HSA. Q and SM competed for the thiol groups of a limited amount of Cys^34^ applied as neat HSA instead of plasma to minimize any side reactions with other plasma proteins. As numerous amino acids of HSA are expected to be prone to alkylation as exemplarily shown for Glu^230^ [12] and Met^329^ [9], a low HSA concentration was applied. After incubation, samples were subjected to pronase-mediated proteolysis to monitor HETE-CP and HETETE-CP simultaneously as measures of their respective HSA-adducts. For comparison, the maximum peak areas of both biomarkers resulting from single incubation (100 μM each) were determined. These values served as a maximum reference (100%) to determine any effective competition that would result in reduced areas. Accordingly, under the assumption of the same alkylating reactivity of SM and Q toward Cys^34^, a decrease to 50% of the reference level would be expected for both biomarkers when incubating with a 1:1-M ratio.

When using 19.6 μM HSA, individual peak areas resulting from co-incubation with 100 μM SM and 100 μM Q were identical to those from separate incubation, thus documenting no competition. This was most likely due to a large extent of additional reactions including simple hydrolysis as well as amino acid alkylations indicating the high reactivity of both vesicants. Therefore, we repeated the incubations using a HSA concentration of 1.96 μM. This is equal to the maximum concentration of free Cys^34^ and was chosen to ensure an effective molar excess of the vesicants when applied at 100 μM concentrations. Results are shown in Table 2.

**Table 2:** Co-incubation to characterize the relative reactivity of SM and Q for Cys^34^ alkylation

Agent incubated and concentration	Relative peak area of HETE-CP [%]	Relative peak area of HETETE-CP [%]	Total of relative peak areas [%]
SM, 100 μM	100	0	100
Q, 100 μM	0	100	100
SM, 100 μM + Q, 100 μM	59 ± 3	79 ± 3	138 ± 6
SM, 100 μM + Q, 10 μM	79 ± 0	12 ± 4	91 ± 4

Relative peak areas were calculated from extracted ion chromatograms of the product ion at *m*/*z* 105.0. Incubations were carried out in triplicate each

*HETE-CP* HSA-derived biomarker obtained after pronase-catalyzed proteolysis of the adduct of HSA and SM, *HETETE-CP* HSA-derived biomarker obtained after pronase-catalyzed proteolysis of the adduct of HSA and Q, *SM* sulfur mustard, *Q* sesquimustard

Resulting reference values of peak areas from triplicate measurements of HETE-CP and HETETE-CP, after individual incubations with 100 μM of SM and Q, respectively, were identical (about 4.85 × 10^5^ cts ± 6%, each) thus supporting the idea of an identical molar yield (identical extent of Cys^34^ alkylation). After co-incubation (100 μM of SM and 100 μM of Q), a reasonably higher yield of HETETE-CP (79 ± 3% of the reference) was found, whereas only 59 ± 3% of the reference was detected for HETE-CP, thus indicating a higher reactivity of Q. The same effect was observed after co-incubation with 100 μM SM and 10 μM Q yielding only 79 ± 1% of the HETE-CP and 12 ± 4% of the HETETE-CP reference. This relative concentration of both vesicants (10:1) was chosen to simulate ratios known from mustard ammunitions [17]. The higher total yield of alkylated CP (sum of HETE-CP and HETETE-CP > 100% when co-incubating with 100 μM each) was presumably due to the higher total vesicant concentration (200 μM instead of 100 μM in single incubation) increasing the product concentration according to the law of mass action.

In summary, Q appears somewhat more reactive than SM (factor 1.33) for Cys^34^ alkylation. This might presumably be due to, e.g., a higher affinity of Q to amino acids close to Cys^34^ resulting in a favorable binding complex or a more precise orientation of the reactive sulfonium ion in a “near-attack” conformation, a higher intrinsic reactivity of the Q sulfonium ion compared to the one of SM, or a smaller extent of side reactions. The important conclusion of our data, however, supports the observation that HETETE-HSA will be produced even though it might be present in much smaller concentrations than SM. This is of high relevance for the possibility of biomarker detection when Q is only found as an impurity or side-product in a mustard mixture.

### Analysis of apo HSA and HETETE-Cys^34^ variants using MD simulations

**Fig. 4:**
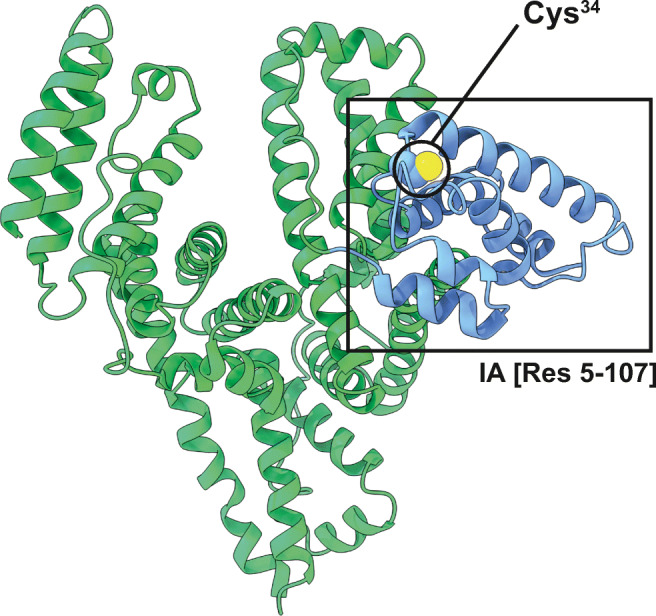
Protein structure of apo HSA (PDB: 1AO6) subdomain IA is highlighted in light blue comprising the Cys^34^ residue that is prone to alkylation by SM and Q resulting in HETE-Cys^34^- and HETETE-Cys^34^-adducts, respectively

As the most abundant protein in human plasma and its importance as a drug carrier and binding partner for many endogenous molecules [42], HSA has been subject to intensive studies using MD simulations [43, 44]. This is also true for the investigation of the role of Cys^34^ that represents the largest pool of free thiol groups in human plasma and shows an unusually low p*K*_a_ of 8.2 [45–47]. It was shown that relatively long simulation times (around 1 μs) are required for adequate structural sampling, but as we are mainly interested in the changes in the local protein environment around Cys^34^, simulation times of 200 ns for apo HSA and 100 ns for the HETETE-adduct were considered appropriate. Previous MD studies have shown that Cys^34^ is only partially solvent accessible and forms a strong hydrogen bond with Tyr^84^ [46]. In the 200-ns simulation of apo HSA, in which the backbone root mean square deviation (RMSD) value stabilizes after around 80 ns, we observed the same phenomenon. Nevertheless, it was also possible to observe and extract various simulation frames in which the S*γ* of Cys^34^ is solvent accessible. We used one such simulation frame to construct the HETETE-adduct in two variants: one with the HETETE moiety pointing into the solvent and one with the HETETE moiety fitting into the groove formed by the two helices adjacent to Cys^34^. The simulation of the “in groove” variant showed a stable backbone RMSD value after about 35 ns and the “in solvent” variant after around 60 ns out of a total of 100 ns simulation time, each. HSA consists of three structurally similar domains (I, II, and III) and each of them is formed by two subdomains (A and B) [48]. Cys^34^ is located in subdomain IA (residues 5–107) (Fig. 4). Other HSA amino acid residues potentially subject to alkylation by SM and Q (E^230^, M^329^) are all solvent exposed without the sterical constraints of Cys^34^.

Visual inspection of the two MD trajectories of the HETETE-adduct and comparison with the apo protein did not yield significant differences in the structure of subdomain IA. The two different “in solvent” and “in groove” starting structures did not result in markedly different trajectories. In all simulations, subdomain IA remained rather rigid with small root mean square fluctuation (RMSF) values per residue compared to other parts of the protein (see ESM Figs. S1–S3).

We observed that the HETETE moiety of the adducted Cys^34^ showed high flexibility and conformational mobility as expected due to the large number of rotatable bonds. This flexibility was, however, steered and restricted by the two helices adjacent to Cys^34^. The HETETE moiety starting at the S*γ* of Cys^34^ moved toward the protein surface while showing an important hydrophobic interaction with the side chain of Leu^42^, that is in almost constant van der Waals contact (average distance 3.8 Å) with the HETETE moiety. The polar end of the HETETE moiety with the terminal hydroxyl group was found to regularly explore the solvent, but for most of the simulation time, it acted as either a hydrogen donor or acceptor interacting with nearby amino acid residues (as H donor: Asp^38^, Lys^41^, Glu^45^, Lys^73^, Thr^76^, Val^77^, Leu^80^; as H acceptor: Lys^41^, Lys^73^, Leu^80^, Arg^81^). By far, the most important of these hydrogen bonding partners are the Glu^45^ carboxyl group and the Lys^41^ amino group. The HETETE hydroxyl group forms a hydrogen bond most often with Glu^45^. The Lys^41^ residue is the second most preferred hydrogen bonding partner apart from the solvent.

**Fig. 5:**
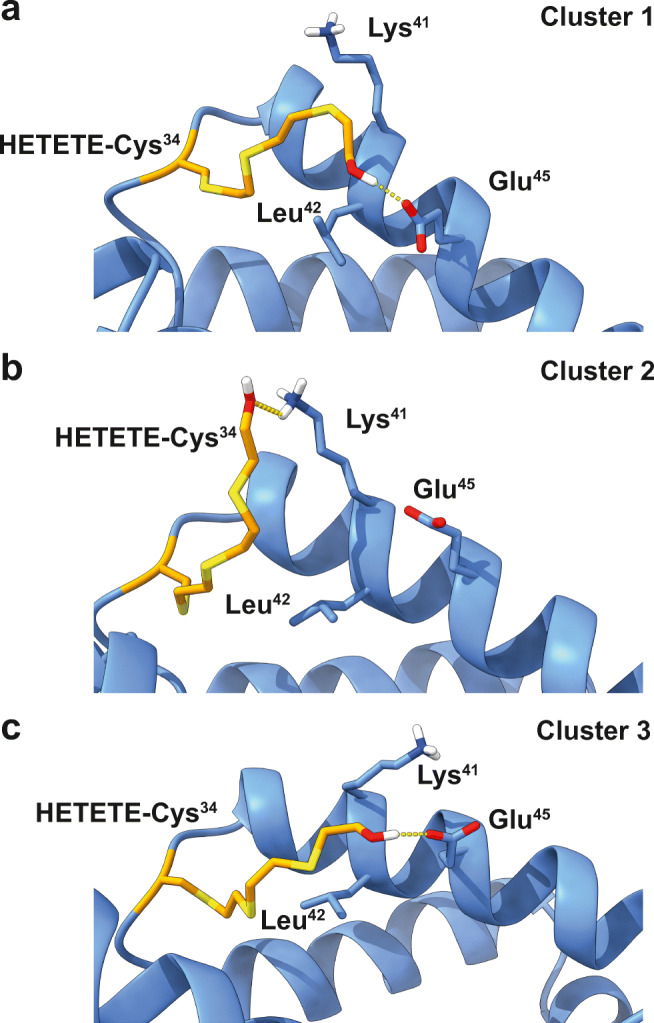
Structural environment around HETETE-Cys^34^ in the three most populated clusters (**a**–**c**) after cluster analysis. Important hydrogen bonding partners (Lys^41^ and Glu^45^) as well as important hydrophobic interaction partners (Leu^42^ and Lys^41^) are highlighted

This observation is also reinforced by cluster analysis employing the method of Daura [49] on those parts of the MD trajectories showing stable backbone RMSD values to reduce the dimensionality of the trajectories containing 10,000 frames each (Fig. 5). The most populated cluster (56% of frames, Fig. 5a cluster 1) shows hydrogen bonding with Glu^45^ and hydrophobic interactions with Lys^41^ and Leu^42^. Despite the polar amino group, the four methylene groups in the Lys^41^ side chain can contribute significantly to hydrophobic interactions as well [50]. The second most populated cluster (27% of frames, Fig. 5b cluster 2) shows hydrogen bonding with the terminal amino group of Lys^45^, while the third most populated cluster (5% of frames, Fig. 5c cluster 3) shows again hydrogen bonding with Glu^45^ and hydrophobic “guidance” by Lys^41^ and Leu^42^. This is in agreement with the hydrogen bonding analysis described above.

In conclusion, the MD simulations show that adduction of Cys^34^ by Q resulting in HETETE-Cys^34^ does not disturb the overall structural rigidity of subdomain IA of HSA, and that within certain steric constraints, the protein can accommodate the quite big HETETE moiety well. HETETE–protein interactions are dominated by hydrogen bonding to Lys^41^ and Glu^45^ and hydrophobic interactions with Lys^41^ and Leu^42^.

### The role of six-membered ring sulfonium ions as reactive species with HSA

**Fig. 6:**
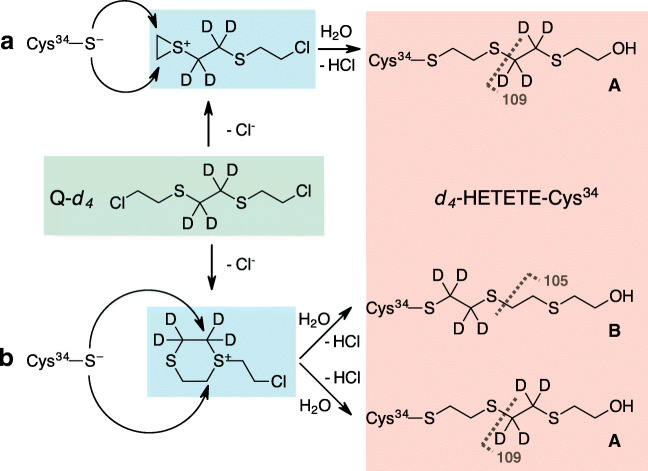
Proposed reaction mechanism for the alkylation of Cys^34^ in HSA by Q. **a** Reaction of Q-*d*_4_ (highlighted in green) with Cys^34^ via the episulfonium ion (highlighted in blue) and hydrolysis of the terminal chlorine atom yielding product A (highlighted in red) producing a deuterated product ion with a nominal mass of 109 in MS/MS fragmentation. **b** Reaction of Q-*d*_4_ (green) with Cys^34^ via the six-membered ring sulfonium ion (blue) and hydrolysis of the terminal chlorine atom yielding two different products—product A as described above and product B yielding the nondeuterated product ion at about *m*/*z* 105 in MS/MS analysis

Three-membered ring episulfonium ions are the only reactive species formed by SM (Fig. 1d). In addition, Q is also able to form six-membered ring sulfonium ions (Fig. 1f). The latter have been observed in aqueous solution as reactive hydrolysis intermediates with significantly higher stability and lifetime than the episulfonium ions [21]. They are also present in significant amounts in old mustard munitions [17]. We were interested if these ions also play a role as reactive intermediates in the reaction of Q with Cys^34^ of HSA and made use of the partially deuterated variant of Q, 1,2-bis(2-chloroethylthio)-*d*_4_-ethane (Q-*d*_4_, Fig. 1c), as a probe. If the reaction between Q-*d*_4_ and Cys^34^ proceeds exclusively via the episulfonium ion, the deuterated ethylene moiety will remain between the two sulfur atoms of Q-*d*_4_ resulting in product A (Fig. 6, product A). A reaction proceeding via the six-membered ring sulfonium ion on the other hand would yield two different products depending on which electrophilic carbon atom the nucleophilic S*γ* of Cys^34^ reacts with (Fig. 6, products A and B). One reaction product is again product A, while the second reaction product B contains the deuterated ethylene moiety in a position directly adjacent to the S*γ* of Cys^34^. Accordingly, mass spectrometric fragmentation by CID should result in a product ion with a nominal mass of 109 derived from product A and in the product ion with a nominal mass of 105 derived from product B (Fig. 6). To elaborate the occurrence of these product ions, *d*_4_-HETETE-CP was subjected to MS/HR MS.

The observation of only very small amounts of the product ion at about *m*/*z* 105 compared with that at about *m*/*z* 109 shows that six-membered sulfonium ions of Q or the half-hydrolyzed Q-chlorohydrin do not play an important role as reactive intermediates in the reaction of Q with Cys^34^ in HSA (data not shown). There are two likely explanations for this observation. Following the hydrolysis of Q in aqueous media by NMR spectroscopy, St. Quintin et al. were able to observe six-membered ring sulfonium ions as long-lived and therefore rather stable intermediates [21]. As high stability and long lifetimes are a result of reduced reactivity, we conclude that episulfonium ions are more reactive and therefore should react with Cys^34^ at higher reaction rates. The second explanation is that the protein environment around Cys^34^ imposes sterical hindrance for the nucleophilic attack of the S*γ* of Cys^34^ on the electrophilic carbon atoms of the bulkier six-membered ring sulfonium ion.

### Potential forensic application

While verification analysis of biomedical samples generally aims to prove or disprove exposure of a victim to a certain chemical agent, there is a growing interest in the possibility to conduct source attribution of a chemical agent. Source attribution means to trace the agent back to a specific synthesis method, reaction batch, specific precursor chemicals, or an existing stockpile. The chemical signatures of environmental samples have been the subject of academic interest [51–54] but have also been used in real cases [55]. So far, biomedical samples have not been considered for source attribution for several reasons: The intact chemical agent and the potential by-products and impurities are normally no longer present in vivo. Biotransformation processes alter compound compositions and impurities that might serve as signatures. In addition, such signatures are typically present in either extremely low concentration or they are biotransformed and excreted from the body seriously impeding their bioanalytical detection.

Sulfur mustards exhibit a complex synthesis and degradation chemistry with a significant number of impurities still being potent vesicants [56]. As described above, Q is an important impurity of SM and its relative amounts depend on the synthesis route, storage conditions, and age. The parallel detection of HETE, HETETE, and potential other adducts of HSA could provide valuable forensic information. In the absence of environmental samples, it might provide clues pointing to the type of mustard used. If a sample of the agent was available, the comparison might allow to conclude whether the exposure was caused by that specific batch of the agent. As adducts with endogenous proteins are regularly detected in the low ppb range, sufficient sensitivity of the method will be challenging, especially if the relative amounts of Q or other blistering impurities are low. Further investigations on human plasma samples of exposed individuals—including retrospective analysis of stored samples—are required for a detailed assessment.

## Electronic supplementary material

ESM 1 (PDF 594 kb).

## Abbreviations

Atr-*d*_3_: Atropine, triple deuterated
BioPT: OPCW biomedical proficiency test
CE: Collision energy
CID: Collision-induced dissociation
CWC: Chemical Weapons Convention
ESI: Electrospray ionization
HETE: Hydroxyethylthioethyl moiety
HETETE: Hydroxyethylthioethylthioethyl moiety
HR MS: High-resolution mass spectrometry
HSA: Human serum albumin
LC: Liquid chromatography
LCt_50_: Median lethal concentration after inhalation
logP: Partition coefficient in octanol/water
LOI: Limit of identification
MD: Molecular dynamics
MS: Mass spectrometry
MS/MS: Tandem mass spectrometry
NMR: Nuclear magnetic resonance
OPCW: Organisation for the Prohibition of Chemical Weapons
Q: Sesquimustard
RMSD: Root mean square deviation
RMSF: Root mean square fluctuation
RT: Room temperature
SM: Sulfur mustard
SRM: Selected reaction monitoring
S_γ_: Sulfur atom of cysteine residue side chain
*t*_R_: Retention time
XIC: Extracted ion chromatogram
μLC: Micro liquid chromatography
