# Supplementary material for: Adduct of the blistering warfare agent sesquimustard with human serum albumin and its mass spectrometric identification for biomedical verification of exposure
Source: Anal Bioanal Chem. 2020 Sep 9;412(28):7723–37. doi: 10.1007/s00216-020-02917-w (PMC7550388; doi:10.1007/s00216-020-02917-w)
Supplement: Supplementary file 1 — (PDF 594 kb). [file 216_2020_2917_MOESM1_ESM.pdf]

Analytical and Bioanalytical Chemistry

Electronic Supplementary Material

Adduct of the blistering warfare agent sesquimustard with human serum albumin and its mass spectrometric identification for biomedical verification of exposure

Marc-Michael Blum, Annika Richter, Markus Siegert, Horst Thiermann, Harald John

1. **Table S1** Product ions of single protonated HETETE-CPF

Structure	Formula	Measured mass	Theoretical mass	Δ [ppm]	Δ [mmu]
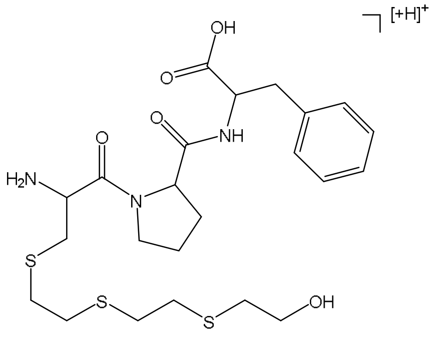	$C_{23}H_{36}O_5N_3S_3$	530.1804	530.1812	-1.4	-0.76
$[M+H-H_2O]^+$	$C_{23}H_{34}O_4N_3S_3$	512.1700	512.1706	-1.2	-0.60
$[M+H-H_2O-NH_3]^+$	$C_{23}H_{31}O_4N_2S_3$	495.1418	495.1441	-4.63	-2.25
$[M+H-2H_2O]^+$	$C_{23}H_{32}O_3N_3S_3$	494.1614	494.1600	2.85	1.37
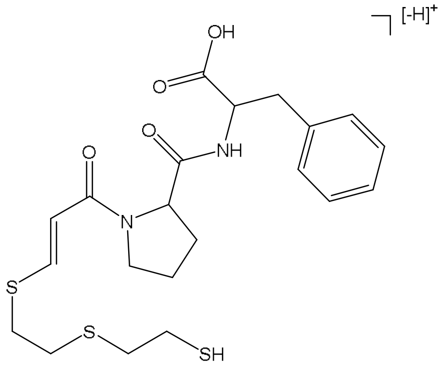	$C_{21}H_{27}O_4N_2S_3$	467.1136	467.1128	1.8	0.85
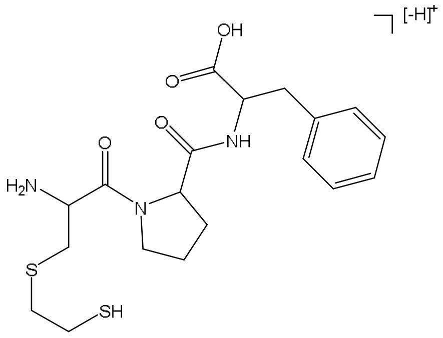	$C_{19}H_{26}O_4N_3S_2$	424.1357	424.1359	-0.64	-0.22
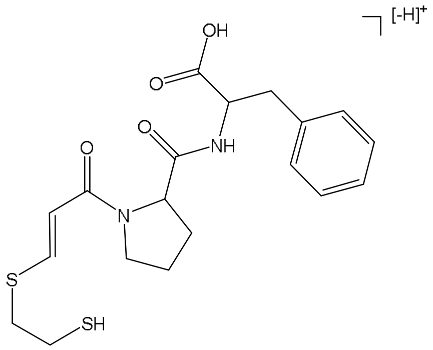	$C_{19}H_{23}O_4N_2S_2$	407.1085	407.1094	-2.17	-0.88

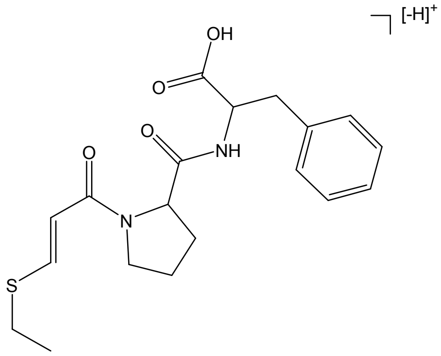	$C_{19}H_{23}O_4N_2S$	375.1366	375.1373	-1.81	-0.70
b_2	$C_{14}H_{25}O_3N_2S_3$	365.1017	365.1022	-1.28	-0.48
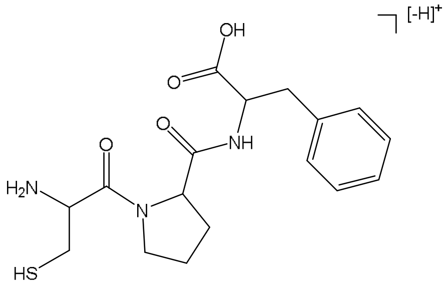	$C_{17}H_{22}O_4N_3S$	364.1317	364.1326	-2.22	-0.85
b_2-NH_3	$C_{14}H_{22}O_3NS_3$	348.0752	348.0756	-1.34	-0.43
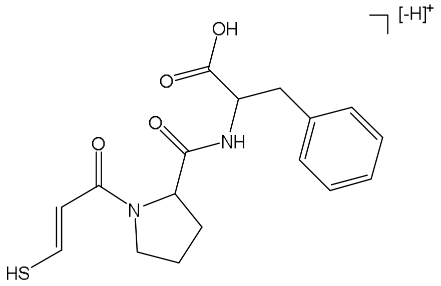	$C_{17}H_{19}O_4N_2S$	347.1061	347.1060	0.22	0.10
a_2	$C_{13}H_{25}O_2N_2S_3$	337.1061	337.1073	-3.34	-1.17
y_2	$C_{14}H_{19}O_3N_2$	263.1385	263.1390	-1.78	-0.52
a_1	$C_8H_{18}ONS_3$	240.0540	240.0545	-2.03	-0.50
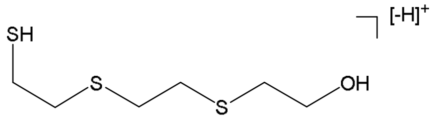	$C_6H_{13}OS_3$	197.0120	197.0123	-1.42	-0.30
y_1	$C_9H_{12}O_2N$	166.0860	166.0863	-1.55	-0.26
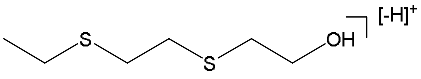	$C_6H_{13}OS_2$	165.0400	165.0402	-1.45	-0.23
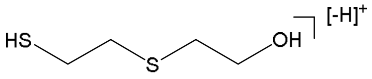	$C_4H_9OS_2$	137.0088	137.0089	-1.27	-0.13
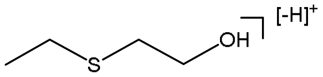	C_4H_9OS	105.0371	105.0369	2.35	0.24
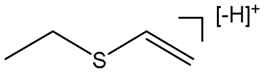	C_4H_7S	87.0268	87.0263	5.53	0.50

Data was extracted from a µLC-ESI MS/HR MS (Orbitrap) run of HETETE-HSA adducts after proteolysis with proteinase K. This reference was produced by incubation of plasma

with Q (100 µM). The corresponding product ion spectrum is shown in Figure 2b. Mass calculation was done using the FreeStyle 1.3 software (Thermo Fisher). Structures represent one possible isomer each.

2. Adding of HETETE-Cys- to the GROMOS96 54a7 force field

Output ITP file of Automated Topology Builder (ATB) v.3.0 for HETETE-Cys (Molecule ID 478917)

```
[ moleculetype ]
; Name   nrexcl
FMOX     3
[ atoms ]
;  nr  type  resnr  resid  atom  cgnr  charge    mass
    1  HS14    1    FMOX    H17    1    0.339   1.0080
    2    OA    1    FMOX     O3    2   -0.479  15.9994
    3  CPos    1    FMOX     C9    3    0.525  12.0110
    4 OEOpt    1    FMOX     O2    4   -0.561  15.9994
    5  CPos    1    FMOX     C8    5    0.240  12.0110
    6    HC    1    FMOX    H16    6    0.077   1.0080
    7  NPri    1    FMOX     N1    7   -0.861  14.0067
    8  HS14    1    FMOX    H18    8    0.358   1.0080
    9  HS14    1    FMOX    H19    9    0.358   1.0080
   10   CH2    1    FMOX     C7   10    0.259  14.0270
   11     S    1    FMOX     S3   11   -0.453  32.0600
   12   CH2    1    FMOX     C6   12    0.153  14.0270
   13   CH2    1    FMOX     C5   13    0.280  14.0270
   14     S    1    FMOX     S2   14   -0.457  32.0600
   15   CH2    1    FMOX     C4   15    0.221  14.0270
   16   CH2    1    FMOX     C3   16    0.219  14.0270
   17     S    1    FMOX     S1   17   -0.431  32.0600
   18   CH2    1    FMOX     C2   18    0.204  14.0270
   19   CH2    1    FMOX     C1   19    0.280  14.0270
   20  OAlc    1    FMOX     O1   20   -0.632  15.9994
   21  HS14    1    FMOX     H1   21    0.361   1.0080
; total charge of the molecule:   0.000
[ bonds ]
;  ai   aj  funct   c0         c1
    1    2    2   0.1000   1.5700e+07
    2    3    2   0.1340   1.0500e+07
    3    4    2   0.1220   2.2843e+07
    3    5    2   0.1540   4.0057e+06
    5    6    2   0.1090   1.2300e+07
    5    7    2   0.1470   8.7100e+06
    5   10    2   0.1530   7.1500e+06
    7    8    2   0.1020   1.7782e+07
    7    9    2   0.1020   1.7782e+07
   10   11    2   0.1830   5.6200e+06
   11   12    2   0.1840   6.9412e+05
   12   13    2   0.1530   7.1500e+06
   13   14    2   0.1840   6.9412e+05
   14   15    2   0.1830   5.6200e+06
   15   16    2   0.1530   7.1500e+06
   16   17    2   0.1830   5.6200e+06
   17   18    2   0.1850   1.0665e+06
   18   19    2   0.1530   7.1500e+06
   19   20    2   0.1430   8.1800e+06
   20   21    2   0.0972   1.9581e+07
[ pairs ]
;  ai   aj  funct  ;  all 1-4 pairs but the ones excluded in GROMOS itp
    1    4    1
    1    5    1
    2    6    1
    2    7    1
    2   10    1
    3    8    1
    3    9    1
    3   11    1
    4    6    1
    4    7    1
    4   10    1
    5   12    1
    6    8    1
    6    9    1
    6   11    1
    7   11    1
    8   10    1
    9   10    1
   10   13    1
   11   14    1
```

```
   12   15    1
   13   16    1
   14   17    1
   15   18    1
   16   19    1
   17   20    1
   18   21    1
[ angles ]
;  ai   aj   ak  funct   angle     fc
    1    2    3    2    104.00   490.00
    2    3    4    2    124.00   730.00
    2    3    5    2    115.00   610.00
    4    3    5    2    121.00   685.00
    3    5    6    2    104.00   490.00
    3    5    7    2    108.00   465.00
    3    5   10    2    111.00   530.00
    6    5    7    2    107.57   484.00
    6    5   10    2    109.50   448.00
    7    5   10    2    115.00   610.00
    5    7    8    2    109.50   425.00
    5    7    9    2    109.50   425.00
    8    7    9    2    106.75   503.00
    5   10   11    2    113.00   545.00
   10   11   12    2    100.00   475.00
   11   12   13    2    113.00   545.00
   12   13   14    2    113.00   545.00
   13   14   15    2    100.00   475.00
   14   15   16    2    113.00   545.00
   15   16   17    2    113.00   545.00
   16   17   18    2    100.00   475.00
   17   18   19    2    113.00   545.00
   18   19   20    2    111.00   530.00
   19   20   21    2    109.50   450.00
[ dihedrals ]
; GROMOS improper dihedrals
;  ai   aj   ak   al  funct   angle     fc
    3    2    4    5    2      0.00   167.36
    5    3   10    7    2     35.26   334.72
[ dihedrals ]
;  ai   aj   ak   al  funct    ph0      cp     mult
    1    2    3    5    1    180.00    16.70    2
    3    5   10   11    1      0.00     5.92    3
    4    3    5   10    1      0.00     1.00    6
    5   10   11   12    1      0.00     2.93    3
   10    5    7    8    1      0.00     3.77    6
   10   11   12   13    1      0.00     2.93    3
   11   12   13   14    1      0.00     5.92    3
   12   13   14   15    1      0.00     2.93    3
   13   14   15   16    1      0.00     2.93    3
   14   15   16   17    1      0.00     5.92    3
   15   16   17   18    1      0.00     2.93    3
   16   17   18   19    1    180.00     1.00    3
   17   18   19   20    1      0.00     5.92    3
   18   19   20   21    1      0.00     1.26    3
[ exclusions ]
;  ai   aj  funct  ;  GROMOS 1-4 exclusions
```

Based on this topology output a new entry was generated in the file aminoacids.rtp in the folder of the forcefield for residue CYSX (using methionine as a starting template)

```
[ CYSX ]
 [ atoms ]
      N     N    -0.31000     0
      H     H     0.31000     0
     CA   CH1     0.00000     1
     CB   CH2     0.24100     2
     SG     S    -0.48200     2
     CD   CH2     0.24100     2
     CE   CH2     0.24100     3
     S2     S    -0.48200     3
     C3   CH2     0.24100     3
     C4   CH2     0.24100     4
     S3     S    -0.48200     4
     C5   CH2     0.24100     4
     C6   CH2     0.24100     5
     O7    OA    -0.64900     5
     H8     H     0.40800     5
      C     C       0.450     6
      O     O      -0.450     6
[ bonds ]
      N     H    gb_2
      N    CA    gb_21
     CA    CB    gb_27
     CA     C    gb_27
```

```
      CB    SG    gb_32
      SG    CD    gb_32
      CD    CE    gb_27
      CE    S2    gb_32
      S2    C3    gb_32
      C3    C4    gb_27
      C4    S3    gb_32
      S3    C5    gb_32
      C5    C6    gb_27
      C6    O7    gb_18
      O7    H8    gb_1
       C     O    gb_5
       C    +N    gb_10
[ angles ]
   ;  ai    aj    ak   gromos type
      -C     N     H     ga_32
      -C     N    CA     ga_31
       H     N    CA     ga_18
       N    CA    CB     ga_13
       N    CA     C     ga_13
      CB    CA     C     ga_13
      CA    CB    SG     ga_16
      CB    SG    CD     ga_4
      SG    CD    CE     ga_16
      CD    CE    S2     ga_16
      CE    S2    C3     ga_4
      S2    C3    C4     ga_16
      C3    C4    S3     ga_16
      C4    S3    C5     ga_4
      S3    C5    C6     ga_16
      C5    C6    O7     ga_13
      C6    O7    H8     ga_12
      CA     C     O     ga_30
      CA     C    +N     ga_19
       O     C    +N     ga_33
[ impropers ]
   ;  ai    aj    ak    al   gromos type
       N    -C    CA     H     gi_1
      CA     N     C    CB     gi_2
       C    CA    +N     O     gi_1
[ dihedrals ]
   ;  ai    aj    ak    al   gromos type
     -CA    -C     N    CA     gd_14
      -C     N    CA     C     gd_44
      -C     N    CA     C     gd_43
       N    CA    CB    SG     gd_34
       N    CA     C    +N     gd_45
       N    CA     C    +N     gd_42
      CA    CB    SG    CD     gd_26
      CB    SG    CD    CE     gd_26
      SG    CD    CE    S2     gd_34
      CD    CE    S2    C3     gd_26
      CE    S2    C3    C4     gd_26
      S2    C3    C4    S3     gd_34
      C3    C4    S3    C5     gd_26
      C4    S3    C5    C6     gd_26
      S3    C5    C6    O7     gd_34
      C5    C6    O7    H8     gd_23
```

The following entry for CYSX was added to the file aminoacids.hdb to account for hydrogen bonding.

```
CYSX    2
1       1       H       N       -C      CA
1       2       H8      O7      C6      C5
```

After constructing the HETETE-moiety using UCSF Chimera the modified residue was named CYSX in the PDB file and the atom names were assigned based on the entry under [atoms] in the aminoacids.rtp file:

CA—CB—SG—CD—CE—S2—C3—C4—S3—C5—C6—O7—H8

3. RMSD fluctuation of MD trajectories

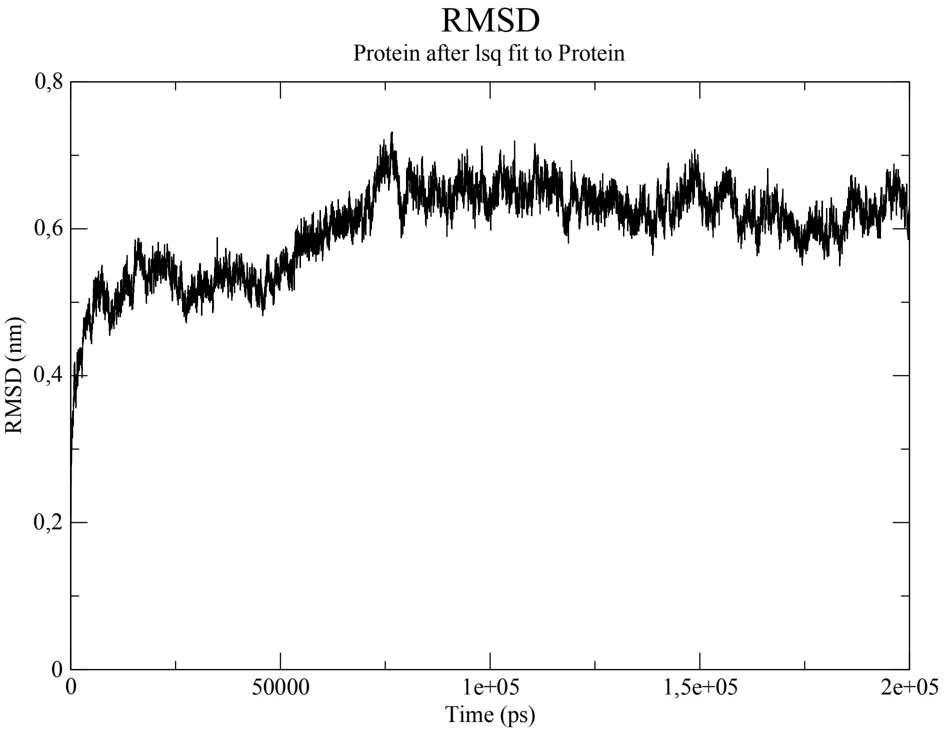

Fig. S1 apo HSA (200 ns)

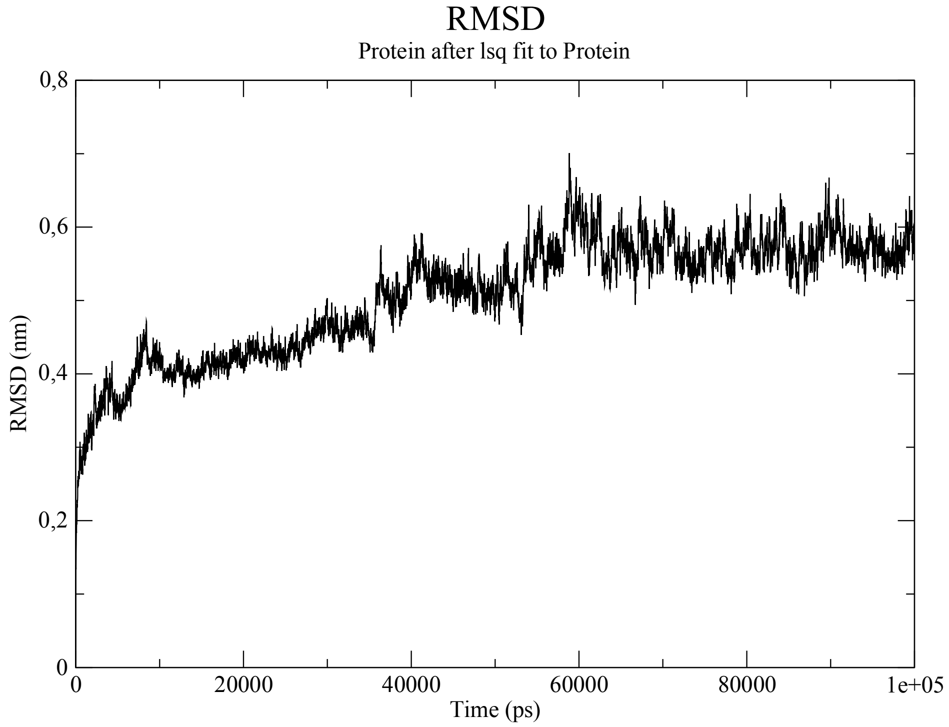

Fig. S2 HETETE-HSA- (100 ns) "in solvent"

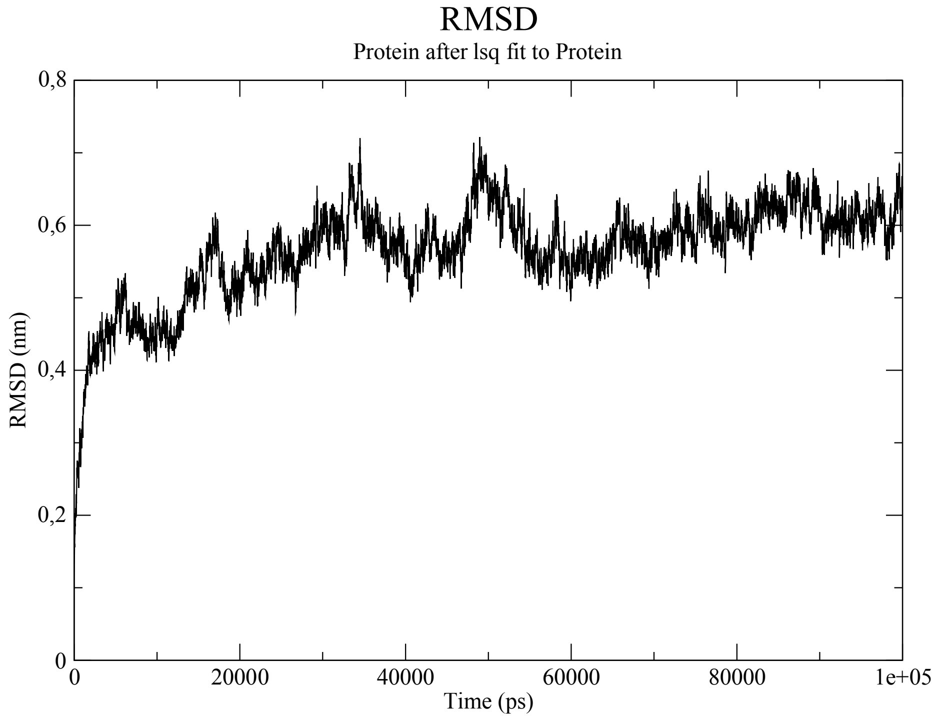

Fig. S3 HETETE-HSA- (100 ns) "in groove"

4. RMSF of residues over the course of the MD simulation with converged RMSD

(C-α group used for RMSD calculation, light grey section depicting subdomain IA)

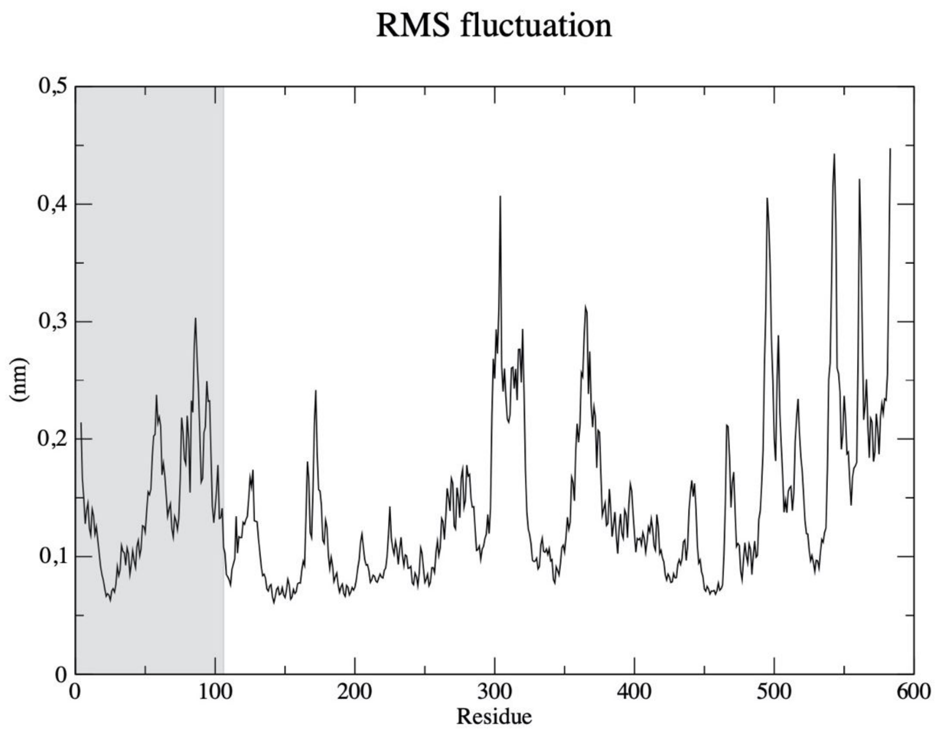

Fig. S4 apo HSA (100 ns – 200 ns)

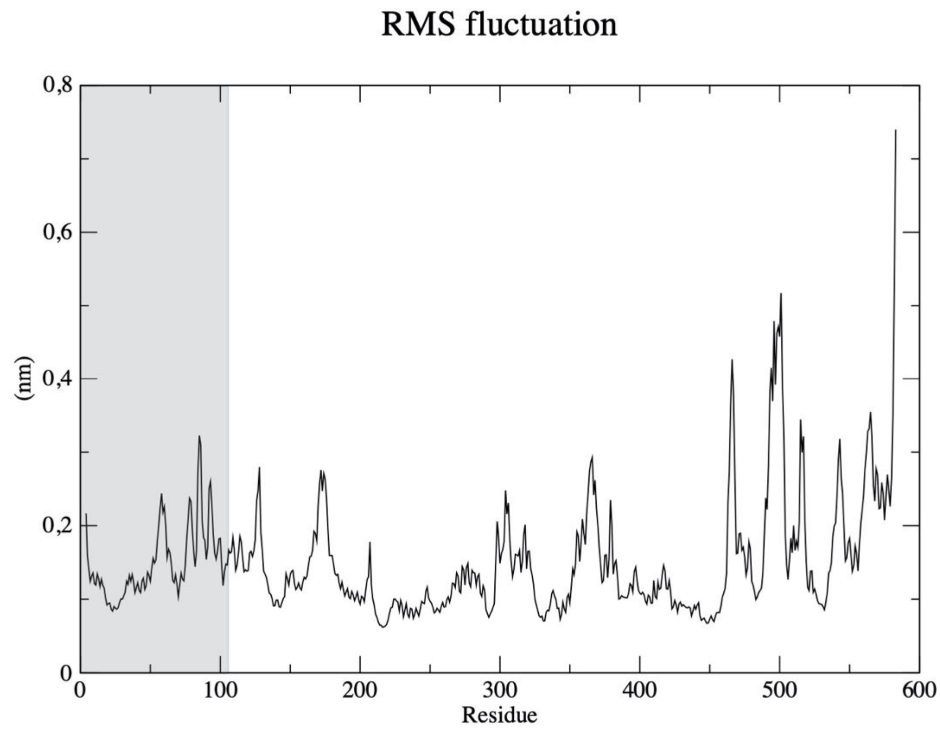

Fig. S5 HETETE-HSA “in solvent” (60 ns – 100 ns)

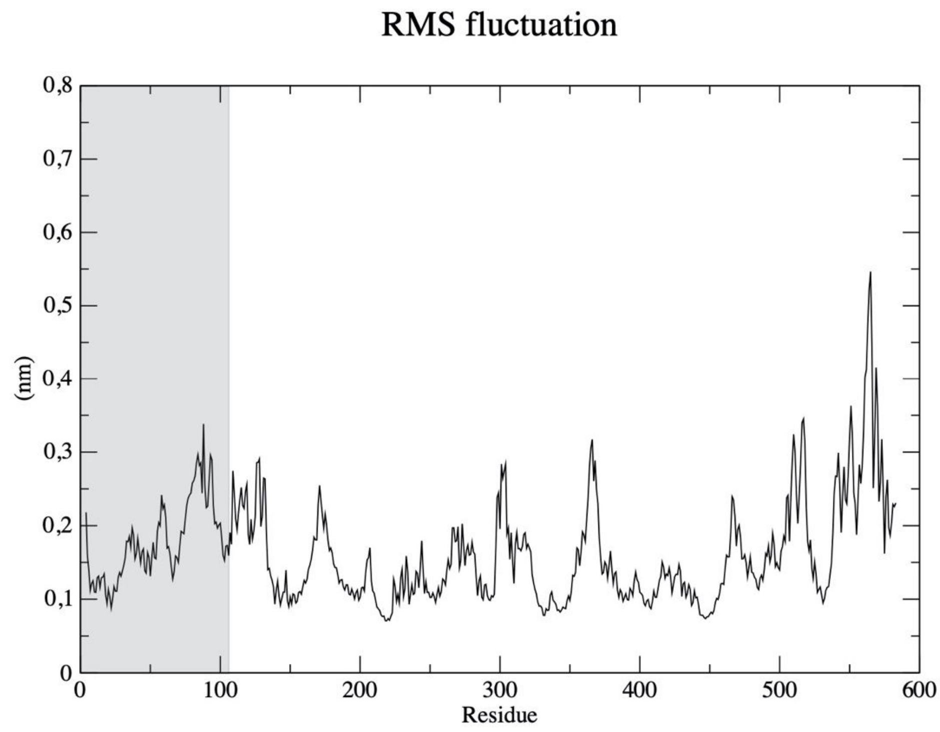

Fig. S6 HETETE-HSA “in groove” (35 ns – 100 ns)
